# Editorial: New insights into spondyloarthritis: from bench to bedside

**DOI:** 10.3389/fimmu.2024.1434616

**Published:** 2024-05-27

**Authors:** Giuseppe Lopalco, Vincenzo Venerito, Gizem Ayan, Michele Maria Luchetti, Maria Sole Chimenti

**Affiliations:** ^1^ Department of Precision and Regenerative Medicine and Ionian Area (DiMePRe-J), University of Bari “Aldo Moro”, Bari, Italy; ^2^ Division of Rheumatology, Department of Internal Medicine, Hacettepe University, Ankara, Türkiye; ^3^ CLINICA MEDICA, Department of Molecular and Biological Sciences, Marche Polytechnic University, Ancona, Italy; ^4^ Department of Internal Medicine, Azienda Ospedaliero Universitaria delle Marche, Ancona, Italy; ^5^ Rheumatology, Allergology and Clinical Immunology, University of Rome Tor Vergata, Rome, Italy

**Keywords:** spondyloarthritis, axial spondylarthritis, cytokines, biologics, advanced treatment, precision medicine

Recent research on spondyloarthritis (SpA) has enlightened the complexities of the disease, providing new insights into its pathogenesis, treatment options, and patient outcomes. This editorial reviews key findings from several significant manuscripts, emphasizing their analytical aspects and implications for clinical practice.

A critical aspect of patient care in SpA is the management of comorbidities, particularly cardiometabolic disorders. Queiro et al. highlight the increased prevalence of cardiometabolic disorders in patients with SpA, including obesity, hypertension, dyslipidemia, and diabetes mellitus, which correlate with increased disease activity and cardiovascular risk. The IL-23/IL-17 axis is identified as a crucial mediator linking inflammation in both SpA and cardiometabolic disorders, with IL-17A playing a key role in promoting inflammation and tissue remodeling. The authors further explore the impact of IL-17 inhibitors, particularly secukinumab, on both SpA symptoms and cardiometabolic profiles. Secukinumab is shown to reduce inflammation markers and potentially decrease cardiovascular risk in SpA patients. The manuscript concludes by underlining the correlation between SpA treatments and cardiometabolic profiles, suggesting IL-17 inhibitors may be preferable for patients with both conditions.

The study, authored by Striani et al. investigates the impact of SARS-CoV-2 infection and vaccination on inflammatory arthritis (IA) patients. It reveals that the prevalence and severity of SARS-CoV-2 infection are similar between IA patients and healthy controls, although hospitalization rates are higher in IA patients. The study also finds that SARS-CoV-2 vaccination does not significantly correlate with IA flare-ups, and vaccination is well tolerated, supporting its recommendation for this patient population. These findings underscore the need for comprehensive care for IA patients, considering both SARS-CoV-2 infection and potential flare-ups. The study enhances our understanding of the bidirectional relationship between SARS-CoV-2 and IA, providing valuable insights for future clinical practice.

The manuscript, by Du et al. investigates the relationship between certain occupational exposures and the risk of developing rheumatoid arthritis (RA) and ankylosing spondylitis (AS). It examines three types of exposures: jobs involving walking or standing, heavy manual or physical work, and shift work. Despite initial indications of a link between heavy manual work and AS, this was not supported after adjusting for confounding factors. The study concludes that the relationship between occupational exposures and RA/AS is complex, requiring further exploration of genetic, environmental, and occupational factors contributing to these diseases.

Moving from bench to bedside Valero-Martínez et al. explores the effectiveness and safety of dual targeted therapy (DTT) for refractory psoriatic arthritis (PsA) and SpA. The study involved 39 DTT combinations administered to 36 patients, with a 69.4% retention rate. Clinical improvements were significant, with 69.4% achieving major clinical improvement and 58.3% achieving remission or low disease activity. The study highlights the coexistence of inflammatory bowel disease in 69% of patients, underscoring the need for multi-domain treatment strategies. It also examines various drug combinations, including TNF inhibitors paired with IL12/23 antagonists or IL17 inhibitors. The study reports only four serious adverse events related to infections, indicating an acceptable safety profile, though further controlled studies are needed to validate these findings and explore long-term outcomes.

One of the key themes emerging from this Research Topic is the pivotal role of genetics in SpA. The manuscript, by Frison et al. provides a comprehensive review of genetic research in SpA and its potential clinical applications. The study emphasizes the role of over 50 susceptibility loci, including HLA-B27, in SpA pathogenesis and its implications for diagnostic biomarkers, polygenic risk scores, and treatment response. The manuscript discusses the limited utility of HLA-B27 testing for diagnostic purposes, remarking that it needs to be combined with clinical and imaging features. It also highlights the potential for genetic research to inform drug development and repurposing, quoting IL-17 blockers influenced by IL23R variants as a key example. The study calls for multidisciplinary collaboration to validate genetic biomarkers and address ethical concerns in precision medicine.

The genetic evidence and therapeutic implications of regulatory T cells (Tregs) in SpA is another intriguing topic. Rodolfi et al. highlight the critical function of Tregs in immune response modulation and maintaining tolerance, discussing the genetic and epigenetic mechanisms that influence Treg stability and function, particularly in the context of AS. The study explores potential therapeutic avenues targeting or enhancing Tregs, including anti-TNF and IL-2 therapies. It concludes by emphasizing the need for deeper research into Treg gene expression, functional variation, and therapeutic interventions, offering potential for adoptive Treg cell therapy.

Pain and its management are major concerns in rheumatology, representing the primary symptom of inflammatory arthropathies needing to be treated. Selmi et al. focus on the role of the JAK/STAT signaling pathway in axial SpA and its implications for pain management. The manuscript emphasizes pain as a critical symptom in axial SpA and highlights the JAK/STAT pathway role in regulating both pro- and anti-inflammatory signaling. Clinical trials of JAK inhibitors have demonstrated significant efficacy in reducing axial SpA disease activity and pain, with *post-hoc* analyses revealing associated improvements in patient-reported outcomes. This review underscores the significance of the JAK/STAT pathway in axial SpA pain mechanisms and its potential for guiding effective treatment strategies, though further studies are needed to explore its impact on different pain types.

In summary, the studies collected in this Research Topic provide valuable insights into the multifaceted nature of SpA, highlighting the need for comprehensive treatment strategies. From managing cardiometabolic comorbidities to exploring genetic, immunological, and therapeutic pathways, these studies advance the understanding of SpA pathogenesis and treatment options. Further research and clinical trials will continue to inform practice, addressing the complex needs of SpA patients.

## Author contributions

GL: Writing – original draft, Writing – review & editing. VV: Writing – original draft. GA: Writing – review & editing. ML: Writing – review & editing. MC: Writing – review & editing.

